# Activation of Sirtuin3 by 6,4′-Dihydroxy-7-methoxyflavanone Against Myoblasts Senescence by Attenuating D-Galactose-Induced Oxidative Stress and Inflammation

**DOI:** 10.3390/nu17203298

**Published:** 2025-10-20

**Authors:** Bingsi Li, Yuxuan Gu, Libing Zhou, Rui Chen, Yiwei Liu, Zexuan Wan, Ziyi Liang, Yukang Wang, Renlei Ji, Zhian Liu

**Affiliations:** 1 Department of Anatomy, Basic Medical College, Xuzhou Medical University, Xuzhou 221004, China; 303101110022@stu.xzhmu.edu.cn (Y.G.); wei_lllll000@163.com (Y.L.); 2 The First Clinical Medical College, Xuzhou Medical University, Xuzhou 221004, China; 202106020114@stu.xzhmu.edu.cn (L.Z.); 202126011019@stu.xzhmu.edu.cn (R.C.); 202126011018@stu.xzhmu.edu.cn (Z.W.); 202126011013@stu.xzhmu.edu.cn (Z.L.); 3 Anesthesia College, Xuzhou Medical University, Xuzhou 221004, China; 202202010413@stu.xzhmu.edu.cn; 4 Department of Cancer Biology, Dana-Farber Cancer Institute, Boston, MA 02215, USA; renlei_ji@dfci.harvard.edu; 5 Department of Biological Chemistry and Molecular Pharmacology, Harvard Medical School, Boston, MA 02215, USA

**Keywords:** DMF, SIRT3, cellular senescence, C2C12 myoblasts, sarcopenia

## Abstract

Background/Objective: Cellular senescence is increasingly recognized as a key mechanism underlying sarcopenia, an age-related muscle disorder with no effective therapeutic. 6,4′-Dihydroxy-7-methoxyflavanone (DMF), a flavonoid isolated from Dalbergia odorifera T. Chen, has shown anti-senescence potential. This study aimed to investigate the protective effects of DMF against myoblasts senescence and elucidate the underlying molecular mechanisms. Method: A cellular model of senescence was established in C2C12 myoblasts using D-galactose (D-gal). The effects of DMF pretreatment were evaluated by assessing senescence phenotypes, myogenic differentiation, and mitochondrial function. The role of Sirtuin3 (SIRT3) was confirmed using siRNA-mediated knockdown. Results: DMF Pre-treatment effectively attenuated D-gal-induced senescence, as indicated by restored proliferation, reduced senescence-associated β-galactosidase activity, decreased DNA damage, and the downregulation of p53, p21*^Cip1/WAF1^* and p16*^INK4a^*. Furthermore, DMF rescued myogenic differentiation capacity, enhancing the expression of Myoblast determination protein 1, Myogenin, Myosin heavy chain and Muscle-specific regulatory factor 4, and promoting myotube formation. Mechanistically, DMF was identified as a SIRT3 activator. It enhanced SIRT3 expression and activity, leading to the deacetylation and activation of the mitochondrial antioxidant enzyme superoxide dismutase 2. This consequently reduced mitochondrial reactive oxygen species, improved mitochondrial membrane potential and ATP production, and suppressed the NF-κB pathway by inhibiting IκBα phosphorylation and p65 acetylation/nuclear translocation. Crucially, all the beneficial effects of DMF—including oxidative stress reduction, mitochondrial functional recovery, anti-inflammatory action, and ultimately, the attenuation of senescence and improvement of myogenesis—were abolished upon SIRT3 knockdown. Conclusions: Our findings demonstrate that DMF alleviates myoblasts senescence and promotes myogenic differentiation by activating the SIRT3-SOD2 pathway, thereby reducing oxidative stress and NF-κB-driven inflammation responses. DMF emerges as a promising therapeutic candidate for sarcopenia.

## 1. Introduction

Sarcopenia is a geriatric syndrome characterized by a progressive and generalized decline in skeletal muscle mass and function associated with aging [1]. It results in reduced mobility and limits independence living and quality of life in the elderly [2]. Cellular aging, known as senescence, is one of the biggest risk factors for skeletal muscle dysfunction [1,3]. Senescent cells exhibit several dramatic changes, including the elevation of proteins that inhibit cell proliferation, increased senescence-associated β-galactosidase (SA-β-gal) activity, and the accumulation of reactive oxygen species (ROS) [4]. Excessive oxidative stress can cause severe damage to DNA and proteins and trigger the secretion of proinflammatory cytokines [4,5]. Previous studies have established C2C12 myoblasts as a widely utilized in vitro model system for investing molecular mechanisms in skeletal muscle biology [6]. Therefore, it is important to explore treatment strategies to prevent or reverse the C2C12 myoblasts senescence.

Among the various cellular organelles implicated in skeletal muscle aging, mitochondria have emerged as central regulators of energy metabolism, redox balance, and apoptotic signaling. Mitochondrial dysfunction is increasingly recognized as a driving force in the progression of sarcopenia. SIRT3 is a member of the sirtuins family of nicotinamide adenine dinucleotide (NAD^+^)-dependent deacetylases [7]. Residing in the mitochondrial matrix, SIRT3 functions as the central regulator of mitochondrial activity. It achieves this by executing reversible lysine deacetylation on a diverse range of metabolic enzymes and proteins [8]. SIRT3 overexpression counteracts p53-driven mitochondrial dysfunction and neurotoxicity in a deacetylase activity-dependent manner [9]. SIRT3 deficiency leads to hyperacetylation of superoxide Dismutase 2 (SOD2) enzyme, resulting in the accumulation of oxidative stress within the mitochondria [10]. In response to calorie restriction, sentrin-specific protease 1-SIRT3 signaling augments the deacetylase activity of SIRT3, promoting mitochondrial fusion [11]. So SIRT3 plays a vital role in the regulation of mitochondria health. Mitochondria have emerged as one of the central regulators of sarcopenia [12]. Hence, SIRT3 might be an important therapeutic target for treating sarcopenia.

Research into the therapeutic potential of nutrients and natural substances represents a pivotal strategy for preventing and managing chronic diseases, as exemplified by their recognized role in modulating immune response and recovery in conditions such as COVID-19 [13]. In the context of combating age-related musculoskeletal disorders like sarcopenia, exploring bioactive natural compounds is particularly promising. Another promising strategy to counteract sarcopenia involves protecting myoblasts from senescence. *Dalbergia odorifera* T. Chen (Leguminosae), a plant used in traditional Chinese medicine, has shown potential. It has been historically employed in the treatment of numerous ailments, such as swelling, necrosis, hematological abnormalities, and rheumatic pain [13]. 6,4′-Dihydroxy-7-methoxyflavanone (DMF), a naturally occurring flavonoid derived from this plant, has been recognized for its ability to suppress oxidative stress, osteoporotic progression, and inflammatory processes [14,15,16]. Previous research demonstrates that DMF augments cellular defense mechanisms against oxidative damage resulting from glutamate-induced cytotoxicity in HT22 neuronal cells [17]. Additional evidence indicates that DMF inhibits osteoclast differentiation by disrupting actin ring formation and suppressing the pit-forming activity of natural osteoclasts [18]. Furthermore, DMF has been shown to attenuate H_2_O_2_-induced cellular senescence in lung fibroblasts through SIRT1 activation [19]. However, the potential involvement of DMF in mitigating aging-related phenotypes within myoblasts, as well as its influence on myogenic differentiation, remains poorly characterized.

Thus, the present study was designed to elucidate the protective role of DMF and its molecular mechanisms against D-gal-induced senescence in C2C12 myoblast cells, along with its potential effects on the process of myogenesis.

## 2. Materials and Methods

### 2.1. Preparation of DMF and D-Gal

**Figure 1:**
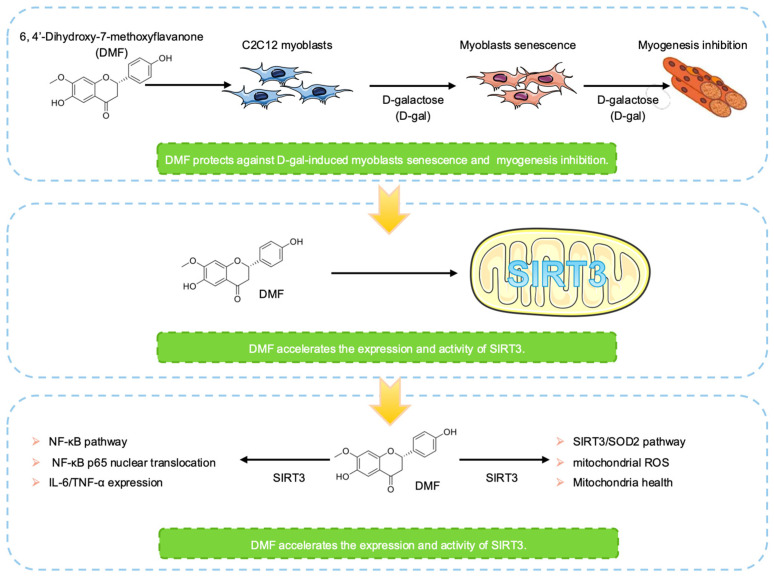
The workflow in this study.

DMF (BBP05065) was obtained from BioBioPha (Kunming, China). DMF was dissolved in Dimethyl sulfoxide (DMSO, D8371, Solarbio, Beijing, China) before used. D-gal (G100367) was obtained from Aladdin (Shanghai, China). D-gal was dissolved in double-distilled water (DW) before used. The final vehicle content was less than 0.01% in each group. DMF and D-gal were used in this study to investigate the effect of DMF on D-gal-induced myoblasts senescence and myogenesis inhibition model. The workflow was show in Figure 1.

### 2.2. Cell Culture

C2C12 myoblasts (a murine cell line, SCSP-505, obtained from Cell Bank/Stem Cell Bank, Chinese Academy of Sciences, Shanghai) were cultured in Dulbecco’s Modified Eagle Medium (DMEM, 12800082, Gibco, Grand Island, NY, USA) containing 10% fetal bovine serum (FBS, 10099141, Gibco) and 1% penicillin/streptomycin (P/S, VC2003, Vicmed, XuZhou, China). C2C12 myoblasts were maintained in differentiation media [DMEM, containing 2% horse serum (HS, 26050070, Gibco) and 1% P/S]. Cells were kept at 37 °C in 5% CO_2_ humidified chambers (Haier, Qingdao, China).

### 2.3. Cell Viability Assay

Proliferation rates and compound toxicity were evaluated via [3-(4,5-dimethylthiazol-2-yl)-5-(3-carboxymethoxyphenyl)-2-(4-sulfophenyl)-2H-tetrazolium, inner salt] test solution (MTS, G3581, Promega, Madison, Wisconsin, USA). Myoblasts (1 × 10^5^ cells/well) in 96-well plates underwent treatment with concentration gradients of D-gal/DMF for varying durations. Fresh MTS working solution (1:20 *v*/*v*) was introduced before 1 h incubation. Absorbance at 490 nm was recorded using a Synergy 2 spectrophotometer (BioTek, Winooski, VT, USA) with Gen5 v2.0 software.

### 2.4. Senescence-Associated β-Galactosidase Staining Assay

Cellular senescence was examined using a commercial SA-β-gal staining kit (C0602, Beyotime, Shanghai, China). Briefly, cells in six-well plates were fixed and then incubated with staining working solution in a dry, CO_2_-free incubators at 37 °C for 6 h. Image acquisition and analysis: Blue-stained SA-β-gal-positive cells were imaged using an Olympus IX73 (Tokyo, Japan) inverted microscope (10×, Japan). For each well, three random, non-overlapping fields were captured. The percentage of SA-β-gal-positive cells was quantified by counting the number of blue cells versus the total number of cells (determined by bright-field imaging) using ImageJ2 2.16.0/1.54p software (NIH) by an investigator blinded to the treatment groups.

### 2.5. Western Blotting Analysis

Collected cells were resuspended in cold RIPA lysis buffer (P0013B, Beyotime) containing 1mM Phenylmethanesulfonyl fluoride (PMSF, ST506, Beyotime). Protein concentration was determined using a bradford proteins assay kit (5000205, Bio-Rad, Hercules, CA, USA). Equal amounts of protein (50 μg) were separated by 12% sodium dodecyl sulfate-polyacrylamide gel and were transferred to Polyvinylidene Fluoride (PVDF) membranes (162-0177, Bio-Rad). Each membrane was incubated with primary antibody at 4 °C overnight, followed by 2 h in secondary antibody at room temperature. Proteins bands were visualized using SuperSignal™ West Pico PLUS Chemiluminescent Substrate (34578, Termo Fisher Scientific, Waltham, MA, USA). Results shown were quantitated using Image J software. Primary antibodies as follows: anti-p53 (60283-2-lg, Research Resource Identifier: AB_2881401, 1:5000, Proteintech, Wuhan, China); anti-p21^Waf1/Cip1^ (28248-1-AP, 1:4000, Proteintech); anti-p16^INK4a^ (ab211542, 1:2000, abcam); anti-phospho-Rb (Ser780) (p-Rb, 8180S, 1:1000, Cell signaling Technology, Beverly, MA, USA); anti-Rb (ab181616, 1:2000, abcam); anti-myoblast determination protein 1 (MyoD, 18943-1-AP, 1:6000, Proteintech); anti-myogenin (Myog, ab124800, 1:1000, abcam); anti-myosin heavy chain (MyHC, sc-376157, 1:2000, Santa Cruz Biotechnology, Inc. Heidelberg, Germany); anti-muscle-specific regulatory factor 4 (MRF4, 11754-1-AP, 1:2000, Proteintech); anti-SIRT3 (10099-1-AP, 1:500, Proteintech); anti-ac-SOD2 (ab137037, 1:10,000, abcam, Cambridge, MA); anti-SOD2 (24127-1-AP, 1:50,000, Proteintech); anti-acetylated-lysine (HA723074, 1:2000, HuaBio, Hangzhou, China); anti-phospho-IκBα (Ser32) (p-IκBα, S0B6000, 1:5000, STARTER, Shanghai, China); anti-IκBα (10268-1-AP, 1:50,000, Proteintech); anti-acetylated nuclear Factor kappa B p65 (ac-NF-κB p65,ab16502, 1:1000, abcam); NF-κB p65 (10745-1-AP, 1:6000, Proteintech); anti-GAPDH (60004-1-Ig, 1:10,000, Proteintech). Secondary antibodies as follows: goat anti-mouse secondary antibody (AS003, 1:5000, Abclonal, Wuhan, China); mouse anti-rabbit secondary antibody (sc-2357, 1:1666, Santa Cruz Biotechnology).

### 2.6. Immunofluorescence

C2C12 myoblasts were grown on gelatin-coated coverslips (22 × 22 mm, China) in six-well plates. After treatments, cells were fixed with 4% paraformaldehyde (VIH100, Vicmed) for 30 min at room temperature (RT). After permeabilization with 0.2% Triton X-100 (T8787, Sigma, St. louis, MO, USA) for 5 min and blocking with 3% Goat Serum Albumin (GSA, WLA067a, Wanleibio, Wuhan, China) for 30 min, the samples were incubated with primary antibodies overnight at 4 °C. The following day, coverslips were incubated with secondary antibodies for 1 h at RT in the dark. Nuclei were counterstained with 1 μg/mL 4′6-diamidino-2-phenylindole (DAPI, S9119, OriLeaf, Suzhou, China). Images were captured using an epifluorescence microscope (Olympus BX43, Tokyo, Japan) and a confocal laser scanning microscope (CLSM, Leica STELLARIS 5, Wetzlar, Germany). Primary antibodies were as follows: anti-MyHC (sc-376157, 1:2000, Santa Cruz); anti-phospho-H2A.X (Ser139) (p-H2A.X, 9718T, 1:250, Cell Signaling Technology); NF-κB p65 (10745-1-AP, 1:100, Proteintech). Secondary antibodies were as follows: SA-ALEXA FLUOR 488 GOAT Anti-Mouse IgG (H + L) Cross-Adsorbed Secondary Antibody (A-11001, 1:1000, Invitrogen); SA-ALEXA FLUOR 568 GOAT Anti-Rabbit IgG (H + L) Cross-Adsorbed Secondary Antibody (A-11011, 1:1000, Invitrogen).

p-H2A.X: Images were captured using a confocal laser scanning microscope (Leica STELLARIS 5). For quantification of DNA damage foci, at least 50 cells per group from three independent experiments were analyzed. The number of discrete p-H2A.X foci per nucleus was counted using ImageJ software (NIH).

MyHC: Images were captured using an epifluorescence microscope (Olympus BX43) with a 20× objective. For myotube analysis, three random fields per group were analyzed. The fusion index was calculated as (number of nuclei within MyHC-positive myotubes containing ≥ 10 nuclei/total number of nuclei) × 100%. Myotube diameter was measured at three random points along the length of each MyHC-positive myotube using Image J.

### 2.7. Detection of ROS

After treatment, cells in six-well were incubated with 10 μM 2′,7′-Dichlorofluorescin diacetate (DCFH-DA, H31224, Aladdin, Shanghai, China) diluted in serum-free DMEM for 30 min at 37 °C in the dark. Following three washes with serum-free DMEM, fluorescence images were immediately captured using an Olympus IX73 inverted microscope (10× objective). Mitochondrial superoxide generation was assessed using MitoSOX Red reagent (G1734, Servicebio, Wuhan, China). Cells were plated in black-walled 96-well plates (Servicebio, China) at a density of 1 × 10^3^ cells per well. Following two washes with 1Xphosphate buffer saline (PBS), cells were loaded with 5 µM MitoSOX Red in recording medium and incubated for 20 min at 37 °C under 5% CO_2_ in darkness. Fluorescence measurements were conducted at 37 °C using a Synergy 2 multi-mode microplate reader (BioTek, USA) equipped with Gen5 v2.0 software, configured for excitation/emission wavelengths of 510/580 nm. The mean fluorescence intensity from three random fields per well was quantified using Image J.

### 2.8. RNA Interference

Myoblasts were transfected with 16 pmol of either SIRT3 small interfering RNAs (SIRT3 siRNA, sc-61556, Santa Cruz Biotechnology) or a fluorescently labeled control siRNA (sc-36869, Santa Cruz Biotechnology) using Lipofectamine 2000 (11668019, Invitrogen) in Opti-MEM reduced-serum medium (31985070, Gibco), following the manufacturer’s instructions. Transfection Efficiency: Transfection efficiency was assessed 24 h post-transfection by quantifying the percentage of cells exhibiting fluorescence from the control siRNA. This analysis consistently revealed a transfection efficiency greater than 80%. The efficacy of SIRT3 knockdown was subsequently validated 72 h post-transfection by Western blot analysis.

### 2.9. SIRT3 Deacetylase Activity Assay

SIRT3 activity was measured using the manufacturer’s instructions using a SIRT3 activity assay kit (ab156067, abcam). Briefly, recombinant SIRT3 was incubated with a reaction mixture composed of assay buffer, Fluoro-Substrate peptide, NAD, developer, and the test compounds. Fluorescence intensity (excitation/emission = 360/460 nm) was recorded at 2-min intervals for 30 min using a microplate reader (Synergy 2 multi-mode microplate reader, BioTek, USA).

### 2.10. Mitochondrial Membrane Potential (MMP) Measurement

Mitochondrial membrane potential was evaluated using a JC-1 assay kit (G515, Servicebio, China) following the manufacturer’s protocol. In brief, after treatment, cells were collected, washed twice with ice-cold PBS, and resuspended in a solution containing 1 mL culture medium and 1 mL JC-1 staining reagent. The suspension was incubated for 20 min at 37 °C in the dark. Following incubation, cells were washed three times with chilled staining buffer and analyzed by flow cytometry (Olympus, Tokyo, Japan). Cellular fluorescence was observed under an Olympus IX73 inverted microscope at 20× magnification.

### 2.11. ATP Production

Cellular ATP content was quantified with a firefly luciferase-dependent ATP detection kit (G4309, Servicebio). Measurements were performed on a Synergy 2 multi-mode microplate reader (BioTek, USA) running Gen5 v2.0 software, in accordance with the manufacturer’s established procedure.

### 2.12. Enzyme-Linked Immunosorbent Assay (ELISA)

The supernatant from C2C12 myoblasts was collected and assayed for IL-6 and TNF-α levels using the ElaBoXTM Mouse ILterleukin-6 (IL-6) ELISA Kit (SEKM-0007, Servicebio) and the ElaBoXTM Mouse TNF-α ELISA Kit (SEKM-0034, Servicebio), respectively, according to the manufacturer’s protocol. Subsequently, the absorbance was measured at a wavelength of 450 nm using a Synergy 2 multi-mode microplate reader.

### 2.13. Statistical Analysis

All data are expressed as the mean ± standard error of the mean (SEM). The sample size (*n*) represents the number of independent biological replicates and is specified in the figure legends. All experiments were performed independently at least three times. Statistical analyses were performed using GraphPad Prism v10.2.3 software. For comparisons between two groups, an unpaired two-tailed Student’s t-test was used. For comparisons among three or more groups under a single factor, one-way analysis of variance (ANOVA) was used, followed by Tukey’s multiple comparison test. For the analysis of cell proliferation across multiple time points, two-way ANOVA followed by Šídák’s multiple comparisons test was applied. A value of *p* < 0.05 was considered statistically significant. Effect sizes with 95% confidence intervals are reported in Supplementary Table S1.

## 3. Results

### 3.1. DMF Attenuates Myoblasts Senescence in C2C12

To investigate the potential therapeutic effects of DMF on aged skeletal muscle, we established an in vitro model of senescent C2C12 myoblasts induced by D-gal. Cells were treated with varying concentrations and different time durations of D-gal (Figure S1). Notably, exposure to 222 mM D-gal for 48 h significantly inhibited cell proliferation (Figure S1A–C). p53, p21*^Cip1/WAF1^* and p16*^INK4a^*, hallmarks of cellular senescence, were upregulated in a concentration- and time-dependent manner after D-gal treatment (Figure S1D,E,H,I). Conversely, p-Rb, hallmarks of cell growth factor, were markedly reduced under these conditions (Figure S1D,E,H,I). Furthermore, the senescent state was corroborated by a significant increase in SA-β-gal-positive cells (Figure S1F,G). Collectively, these data demonstrated that treatment with 222 mM D-gal for 48 h represents an optimal approach for establishing a robust cellular senescence model. The p-H2A.X, a DNA damage marker, provided additional validation of this model (Figure S1J,K).

**Figure 2:**
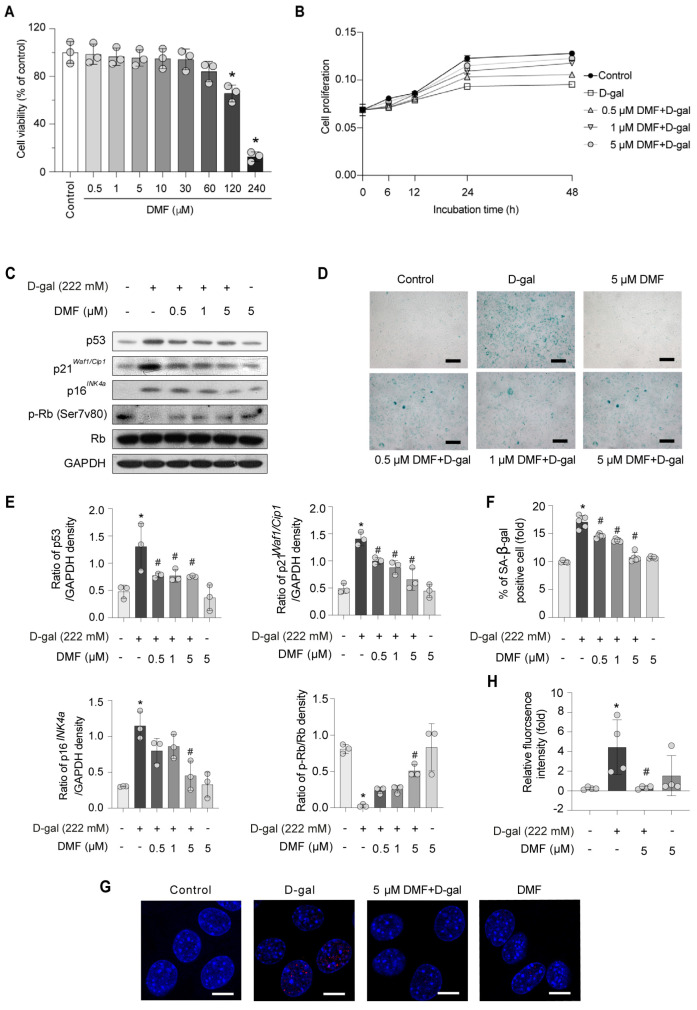
DMF attenuates D-gal-induced premature senescence in C2C12 myoblasts. (**A**) C2C12 myoblasts were treated with indicated concentrations DMF for 12 h, followed by treatment with or without 222 mM D-gal for an additional 48 h to induce premature senescence. Cell viability (**A**) and cell proliferation (**B**) were examined by MTS assay. (**C**) p53, p21*^Waf1/Cip1^*, p16*^IN4a^*. p-Rb (Ser7v80), Rb and GAPDH were analyzed by western blotting. (**D**) Representative images of SA-β-gal staining. Scale bar, 200 μm. (**E**) Quantitative analysis of protein levels from (**C**). (**F**) Quantification of the SA-β-gal-positive cell from (**D**). (**G**) Representative immunofluorescence images of p-H2A.X (red). Nuclei were counterstained with DAPI (blue). Scale bar, 10 μm. (**H**) Quantitative analysis of the p-H2A.X fluorescence intensity from (**G**). Data are shown as the mean ± SEM from three independent experiments (*n* = 3). ∗ *p* < 0.05 versus the control group, # *p* < 0.05 versus D-gal-only treatment group.

To evaluate the potential cytotoxicity of DMF, myoblasts were pre-treated varying concentrations of DMF for 12 h (Figure 2A) prior to exposure to 222 mM D-gal (Figure 2B), followed by assessment with an MTS assay. As shown in Figure 2A, 0–30 μM DMF have no significant cytotoxicity. Pretreatment with 0.5–5 μM DMF ameliorated the growth inhibitory effect induced by D-gal treatment (Figure 2B). Consistent with these findings, the expression levels of aging-related markers and cell growth factor showed comparable responses following DMF treatment (Figure 2C,E). Meanwhile, DMF pretreatment resulted in a concentration-dependent reduction in SA-β-gal positive area (Figure 2D,F). These results suggest that DMF exerts an anti-aging effect and 5 μM DMF treatment have a best effect. The expression of p-H2A.X further verified these observations (Figure 2G,H).

### 3.2. DMF Promotes Myogenesis by Alleviating D-Gal-Induced Senescence in Myoblasts

**Figure 3:**
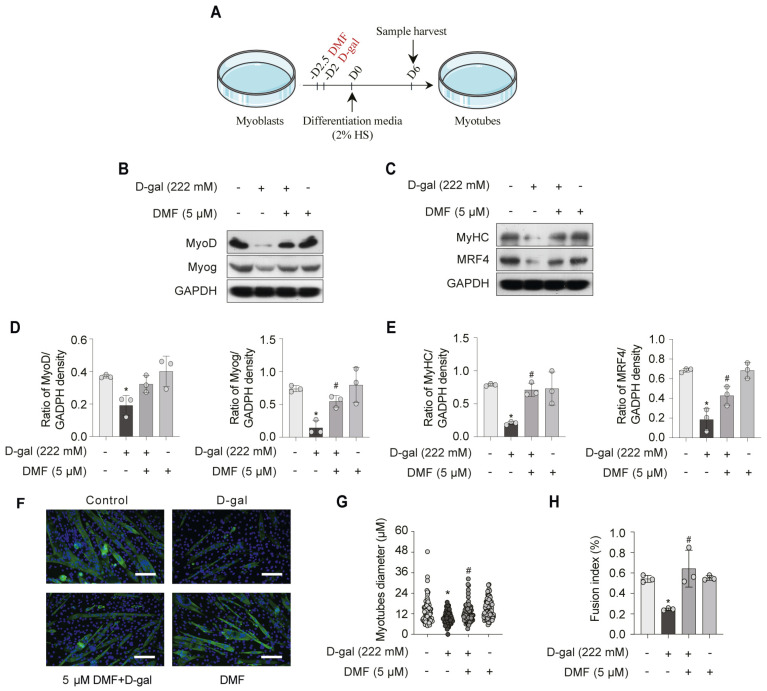
DMF promotes myotube formation in senescent C2C12 myoblasts. (**A**) Schematic diagram of the experimental timeline. C2C12 myoblasts were pre-treated with 5 μM DMF for 12 h, followed by treatment with or without 222 mM D-gal for 48 h to induce senescence. Cells were then switched to differentiation medium for 6 days (D6) to induce myogenic differentiation. Protein levels of early markers (MyoD and Myog) (**B**) and late differentiation markers (MyHC and MRF4) (**C**) were analyzed by western blotting. (**D**) Quantitative analysis of MyoD and Myog protein levels from (**B**). (**E**) Quantitative analysis of MyHC and MRF4 protein levels from (**C**). (**F**) Representative immunofluorescence images of MyHC (green). Nuclei were counterstained with DAPI (blue). Scale bar, 100 μm. (**G**) Quantification of myotubes diameter from (**F**). (**H**) Quantification of the fusion index from (**F**), defined as the percentage of nuclei contained within MyHC-positive myotubes (with ≥10 nuclei). Data are shown as the mean ± SEM from three independent experiments (*n* = 3). ∗ *p* < 0.05 versus the control group, # *p* < 0.05 versus D-gal-only treatment group.

To assess myogenic differentiation, we examined the expression of key markers, including the early factors (MyoD and MyoG) and late factors (MyHC and MRF4). We found that these differentiation markers were suppressed following D-gal-induced senescence in myoblasts (Figure 3B–E). In contrast, pre-treatment with DMF significantly up-regulated their expression (Figure 3B–E). Moreover, DMF pre-treatment led to an increase in myotubes diameter and a higher fusion index (Figure 3F–H). These findings suggest that DMF exerts an anti-aging effect by attenuating senescence in myoblasts and facilitating myotubes development.

### 3.3. DMF, a SIRT3 Activator, Enhances SOD2 Activity and Inhibits Mitochondrial ROS (mtROS) in C2C12 Myoblasts

**Figure 4:**
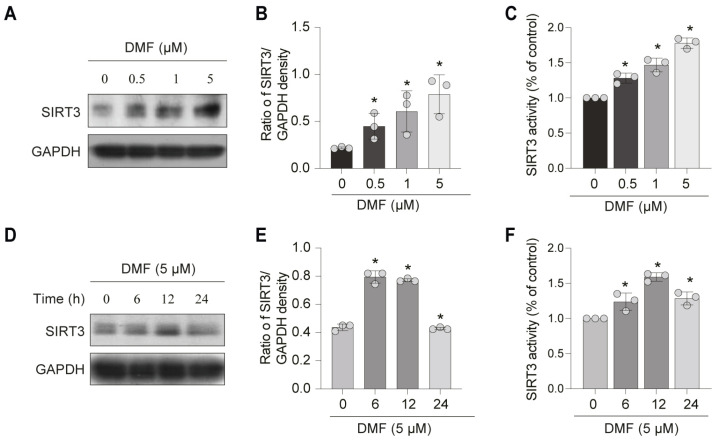
DMF promotes SIRT3 expression in C2C12 myoblasts. (**A**–**C**) C2C12 myoblasts were treated with 0.5–5 μM DMF for 12 h. (**D**–**F**) C2C12 myoblasts were treated with 5 μM DMF for 0–24 h. (**A**,**D**) SIRT3 and GAPDH were analyzed by western blotting. (**B**,**E**) Quantitative analysis of protein levels from (**A**,**D**). (**C**,**F**) SIRT3 activity was used a SIRT3 fluorometric kit. Data are shown as the mean ± SEM from three independent experiments (*n* = 3). ∗ *p* < 0.05 versus control (Con).

**Figure 5:**
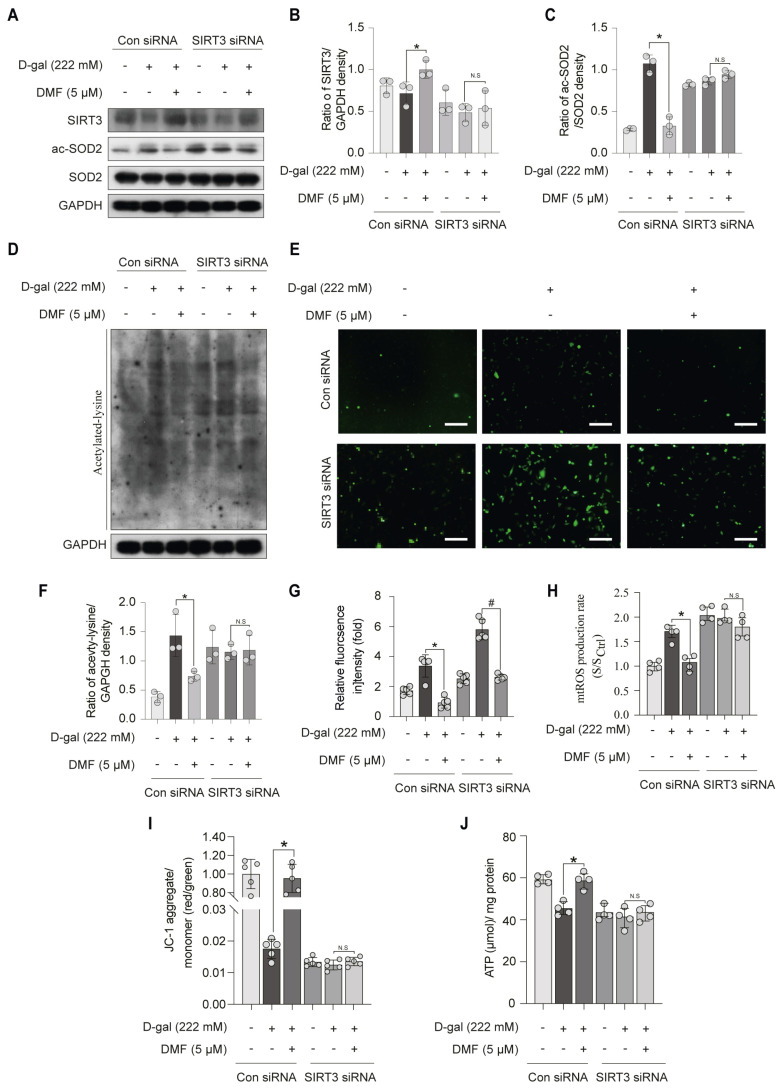
DMF alleviates ROS expression by activating SIRT3/SOD2 pathway. C2C12 myoblasts were pre-transfected with a control siRNA or SIRT3 siRNA (16 pmol, 18 h) in the present or absence of DMF (5 μM, 12 h) before D-gal (222 mM, 48 h) treatment. (**A**) SIRT3, ac-SOD2, SOD2 and GAPDH were analyzed by western blotting. (**B**,**C**) Quantitative analysis of protein levels from (**A**). (**D**) Acetylated lysine and GAPDH were analyzed by western blotting. (**E**) Expression of ROS was assessed by DCFH-DA fluorescent staining (green). Scale bar, 200 μm. (**F**) Quantitative analysis of protein levels from (**D**). (**G**) Quantification of ROS levels from (**E**). (**H**) mitochondrial ROS generation was assessed using MitoSOX Red reagent. (**I**) Mitochondrial membrane potential was evaluated using a JC-1 assay kit. (**J**) Cellular ATP content was quantified with a firefly luciferase-dependent ATP detection kit. Data are shown as the mean ± SEM from three independent experiments (*n* = 3). ∗ *p* < 0.05 versus D-gal with control (Con) siRNA, # *p* < 0.05 versus D-gal with SIRT3 siRNA, N.S: not significant.

To investigate the regulatory mechanism underlying the anti-aging effect of DMF, we examined its impact on SIRT3, a key mitochondrial deacetylase implicated in aging. C2C12 myoblasts were treated with varying concentrations of DMF for 12 h. DMF up-regulated SIRT3 protein expression and activity in a concentration-dependent manner (Figure 4A–C). Optimum SIRT3 induction was achieved at 5 μM DMF. After treatment with 5 μM DMF, the protein expression and activity of SIRT3 peaked at 12 h and declined by 24 h (Figure 4D–F). To determine whether DMF attenuates SOD2 acetylation via SIRT3, we performed siRNA-mediated knockdown of SIRT3 in the context of D-gal-induced stress. As shown in Figure 4A–C, Pre-treatment with DMF attenuated SOD2 acetylation through up-regulation of SIRT3. This effect was markedly attenuated upon SIRT3 knockdown using siRNA in C2C12 myoblasts (Figure 5A–C). Consistent results were observed in the overall levels of acetylated lysine in total cellular proteins (Figure 5D,F). These data indicated that DMF enhanced the deacetylase activity of SIRT3 (Figure 5D,F). DCFH-DA fluorescent staining demonstrated that DMF significantly suppressed ROS generation compared to the D-gal-treated group (Figure 5E,G). However, the protection effect of DMF was abolished upon SIRT3 inhibition (Figure 5E,G). Mitochondrial-specific ROS, as measured by MitoSOX Red fluorescence, followed the same pattern (Figure 5H). Furthermore, DMF improved mitochondrial function, as indicated by the recovery of mitochondrial membrane potential (JC-1 assay, Figure 5I) and increased ATP production (Figure 5J). These beneficial effects were also SIRT3-dependent. Collectively, these results indicate that DMF enhances SIRT3 expression and activity, leading to SOD2 deacetylation, reduced mitochondrial ROS accumulation, and improved mitochondrial function.

### 3.4. DMF Attenuates D-Gal-Induced NF-κB Activation in a SIRT3-Dependent Manner

**Figure 6:**
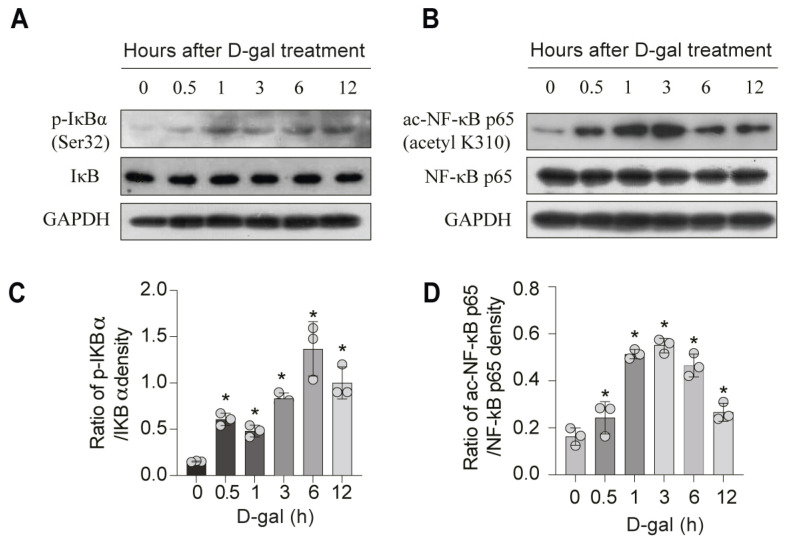

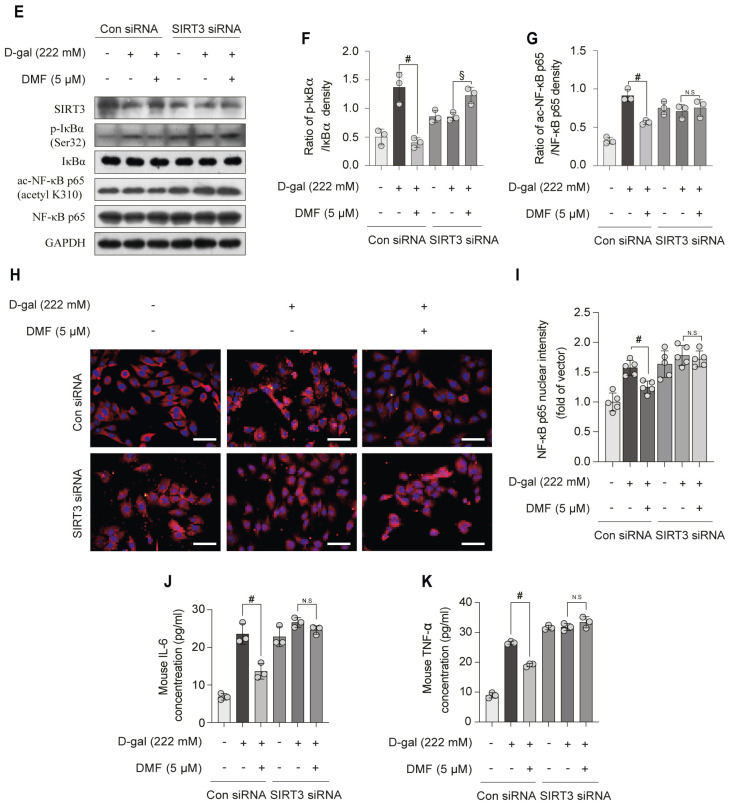
DMF inhibits NF-κB activation in C2C12 myoblasts. (**A**–**D**) C2C12 myoblasts were treated with 222 mM D-gal with 0.5–12 h. (**A**) p-IκBα, IκBα and GADPH were analyzed by western blotting. (**B**) ac-NF-κB p65, NF-κB p65 and GADPH were analyzed by western blotting. (**C**) Quantitative analysis of protein levels from (**A**). (**D**) Quantitative analysis of protein levels from (**B**). (**E**) SIRT3, p-IκBα, IκBα, ac-NF-κB p65, NF-κB p65 and GADPH were analyzed by western blotting. (**F**,**G**) Quantitative analysis of protein levels from (**E**). (**H**) Representative immunofluorescence images of p65 subunit of NF-κB (red) distribution. Nuclei were counterstained with DAPI (blue). Scale bar, 50 μm. (**I**) Quantitative analysis of NF-κB p65 fluorescence intensity in nuclear distribution from (**H**). (**J**,**K**) The secretion of IL-6 and TNF-α were analyzed by ELISA. Data are shown as the mean ± SEM from three independent experiments (*n* = 3). ∗ *p* < 0.05 versus control, # *p* < 0.05 versus D-gal with Con siRNA, § *p* < 0.05 versus D-gal treatment with SIRT3 siRNA, N.S.: not significant.

NF-κB represents a central transcription factor involved in the inflammatory response. Activation of the NF-κB pathway induces the transcription of multiple genes, including those encoding pro-inflammatory cytokines, which have been closely linked to the pathogenesis of age-related diseases [3]. To investigate the effect of D-gal exposure on NF-κB pathway, we assessed the ac-NF-κB p65 and p-IκBα in C2C12 myoblasts. Treatment with 222 mM D-gal resulted in a time-dependent increase in p-IκBα, with a significantly elevation observed between 1–12 h (Figure 6A,C). NF-κB p65 acetylation peaked around 0.5–3 h and gradually declined thereafter (Figure 6B,D). Pre-treatment with DMF markedly attenuated these increases (Figure 5E–G). Notably, this inhibitory effect of DMF was substantially abolished following SIRT3 knockdown via siRNA in myoblasts (Figure 6E–G). Moreover, DMF significantly suppressed D-gal-induced nuclear translocation of NF-κB p65, an effect that was also reversed by SIRT3 knockdown (Figure 6H,I). Consistent with these findings, DMF pretreatment reduced the secretion of the pro-inflammatory cytokines IL-6 and TNF-α induced by D-gal (Figure 6J,K). Collectively, these results demonstrate that DMF suppressed D-gal-induced NF-κB activation by suppressing IκBα phosphorylation and NF-κB p65 acetylation in a SIRT3 dependent manner.

## 4. Discussion

In this study, we employed a pre-treatment strategy to investigate the potential of DMF in preventing or delaying oxidative stress-induced senescence in C2C12 myoblasts. This approach is particularly relevant for evaluating DMF as a nutraceutical or dietary supplement, where the goal is often to bolster cellular defenses against impending stressor, rather than reversing established damage. Under this paradigm, we demonstrated that pre-emptive exposure to DMF effectively shielded myoblasts from D-gal-induced senescence, as evidenced by the attenuation of key senescence markers, including growth arrest, SA-β-gal activity, DNA damage, and impaired differentiation.

Aging is a complex biological process driven by multifaceted mechanisms including oxidative stress, genomic instability, and chronic inflammation, all of which contribute to the functional decline of tissues and increase susceptibility to age-related diseases [20,21,22]. Sarcopenia is an aging-related skeletal muscle disorder disease which is manifested as accelerated loss of muscle mass, decreased muscle strength and muscle dysfunction [1]. Accumulating evidence suggests that cellular senescence and the associated secretory phenotype impose substantial burdens on muscle homeostasis, with oxidative stress being a central contributor to it pathophysiology [5,23].

Mitochondrial integrity is essential for maintaining normal skeletal muscle function due to the high energy demand of muscle tissue [24]. Previous study reports that mitofusin 2 deficiency links sarcopenia and impairs autophagy to activation of mitophagy [25]. SIRT3, a NAD^+^-dependent deacetylase predominantly localized in the mitochondrial matrix, plays an important role in various mitochondrial metabolism, including the urea cycle, fatty acid oxidative, ROS detoxification, mitochondrial [7]. Qiu et al. (2010) found activation SIRT3 during calorie restriction reduces oxidative stress through activation of the mitochondrial antioxidant enzyme SOD2 [26]. Upregulation of SIRT3 deacetylase activation promotes both aerobic glycolysis and oxidative phosphorylation (OXPHOS), as well as mitochondrial fusion, via the sentrin-specific protease 1/SIRT3 pathway [11]. We speculated that SIRT3 may be a promising therapeutic target for sarcopenia. In our previous work, we established that 20–80 μM DMF acts as activator of SIRT1 [19]. Intriguingly, compared to its effect on SIRT1, a significantly lower concentration of DMF (0.5–5 μM) is sufficient to upregulate SIRT3. Our findings suggest that DMF may be a more potent activator of SIRT3 than SIRT1.

Having established that pre-treatment with DMF alleviates oxidative stress—a key trigger of both senescence and inflammatory responses—our finding position DMF as a promising candidate for preventive intervention against sarcopenia. The NF-κB pathway is a central mediator of inflammation and can be regulated by post-translational modifications such as acetylation. According to a recent study, Inorganic trace elements mitigate sarcopenia pathology by promoting NF-kB deacetylation to inhibit M1 polarization, which ultimate reduces the expression of inflammatory factors [27]. NF-κB activation can be achieved through two distinct mechanisms: the canonical IκB-dependent pathway, which involves phosphorylation and subsequent degradation of IκB, or the non-canonical IκB-independent pathway, which relies on post-translational modifications—such as acetylation—of Rels proteins, including the NF-κB p65 subunit [28]. Since SIRT3, the key mediator of DMF’s effect in our study, is known to influence cellular acetylation status, we hypothesized that DMF might also affect NF-κB acetylation. Recent studies suggest that modulation of NF-κB acetylation may represent a viable therapeutic strategy.

Natural compound, particularly plant-derived flavonoids, have garnered increasing attention for their potential in preventing age-related muscle loss due to their favorable safety profiles and multi-target activities [29,30]. DMF, a flavonoid isolated from Dalbergia odorifera T. Chen, has antioxidant, anti-inflammatory, and other pharmacological effects [17,18]. Our findings reveal that DMF upregulates both the expression and activity of SIRT3. In contrast, knockdown of SIRT3 by siRNA abolished the protective effects of DMF, resulting in renewed ROS accumulation and NF-κB pathway activation.

It is important to acknowledge a limitation of the present study. While the C2C12 murine myoblast model has provided robust and consistent data that form a solid foundation for our conclusions, the findings warrant broader validation. A crucial next step will be to investigate the effects of DMF in additional cell models, particularly in human myoblasts, to better assess its translational potential.

## 5. Conclusions

In summary, our findings demonstrate that pre-treatment with DMF effectively attenuates oxidative stress-induced senescence in C2C12 myoblasts. The protective mechanism is primarily mediated through the upregulation of SIRT3, which subsequently suppresses ROS generation and inhibits the NF-κB-mediated inflammatory response. These findings position DMF as a promising nutraceutical candidate for preventing myoblasts senescence. However, it is crucial to note that this research is limited to a cell-based model. Therefore, future work is indispensable to validate these findings in vivo. Key next steps should include pharmacokinetic studies in mice to determine the oral bioavailability and safety of DMF, followed by efficacy assessments in aged mice models of sarcopenia, focusing on critical functional outcomes such as muscle strength, endurance, and mass. These studies are essential to fully appraise the translational potential of DMF in combating age-related muscle wasting.

## Data Availability

The data used to support the findings of this study are available from the corresponding author upon request.

## Supplementary Materials

The following supporting information can be downloaded at: https://www.mdpi.com/article/10.3390/nu17203298/s1, Figure S1: D-gal induces C2C12 myoblasts premature senescence. (**A**,**B**) Cell viability and cell proliferation were examined by MTS assay. (**C**) Cell morphology was observed using light microscopy. Scale bar, 200 μm. (**D**) The protein expression was analyzed by western blotting. (**E**) Quantification of protein levels from (**D**). (**F**,**G**) Representative images of SA-β-gal staining of C2C12 myoblasts and SA-β-gal positive area%. Scale bar, 200 μm. (**H**) The protein expression was analyzed by western blotting. (**I**) Quantification of protein levels from (**H**). (**J**) Expression of p-H2A.X (red) was assessed by immunofluorescence staining, along with nuclear counterstaining using DAPI (blue). Scale bar, 5 μm. (**K**) Quantification of p-H2A.X levels from (**J**). Data are shown as the mean ± SEM from three independent experiments (*n* = 3). ∗ *p* < 0.05 versus control. Table S1: Statistical Tests, *p* Values, and Effect Sizes (95% CI) for Figures C, D, F, G, I, J, and K.

## Abbreviations

The following abbreviations are used in this manuscript:

ac-NF-κB p65	acetylated nuclear Factor kappa B p65
ANOVA	analysis of variance
Con	Control
D6	6 days
DMF	6,4′-Dihydroxy-7-methoxyflavanone
D-gal	D-galactose
DMSO	Dimethyl sulfoxide
DW	double-distilled water
DAPI	4′6-diamidino-2-phenylindole
d/D	Day
DMEM	Dulbecco’s Modified Eagle Medium
DCFH-DA	2′,7′-Dichlorofluorescin diacetate
ELISA	Enzyme-linked immunosorbent assay
FBS	fetal bovine serum
GSA	Goat Serum Albumin
HS	horse serum
IL-6	ILterleukin-6
MTS	[3-(4,5-dimethylthiazol- 2-yl)-5-(3-carboxymethoxyphenyl)-2-(4-sulfophenyl)-2H-tetrazolium, inner salt] test so-lution
MyoD	Myoblast determination protein 1
MyoG	Myogenin
MyHC	Myosin heavy chain
MRF4	Muscle-specific regulatory factor 4
MMP	Mitochondrial membrane potential
NAD	Nicotinamide adenine dinucleotide
N.S.	not significant
P/S	penicillin/streptomycin
PMSF	Phenylmethanesulfonyl fluoride
PVDF	Polyvinylidene fluoride
p-Rb	Phospho-Rb
p-H2A.X	Phospho-H2A histone family member X
p-IκBα	Phosphorylated IκBα
PBS	phosphate buffer saline
ROS	Reactive oxygen species
mtROS	mitochondrial ROS
RT	Room temperature
SA-β-gal	Senescence-associated β-galactosidase
SEM	standard error of the mean
siRNAs	Small interfering RNAs
SOD2	Superoxide Dismutase 2
SIRT3	Sirtuin3
