# Supplementary material for: Activation of Sirtuin3 by 6,4′-Dihydroxy-7-methoxyflavanone Against Myoblasts Senescence by Attenuating D-Galactose-Induced Oxidative Stress and Inflammation
Source: Nutrients. 2025 Oct 20;17(20):3298. doi: 10.3390/nu17203298 (PMC12567291; doi:10.3390/nu17203298)
Supplement: Supplementary file 1 [file nutrients-17-03298-s001.zip › Figure S1-nutrients.pdf]

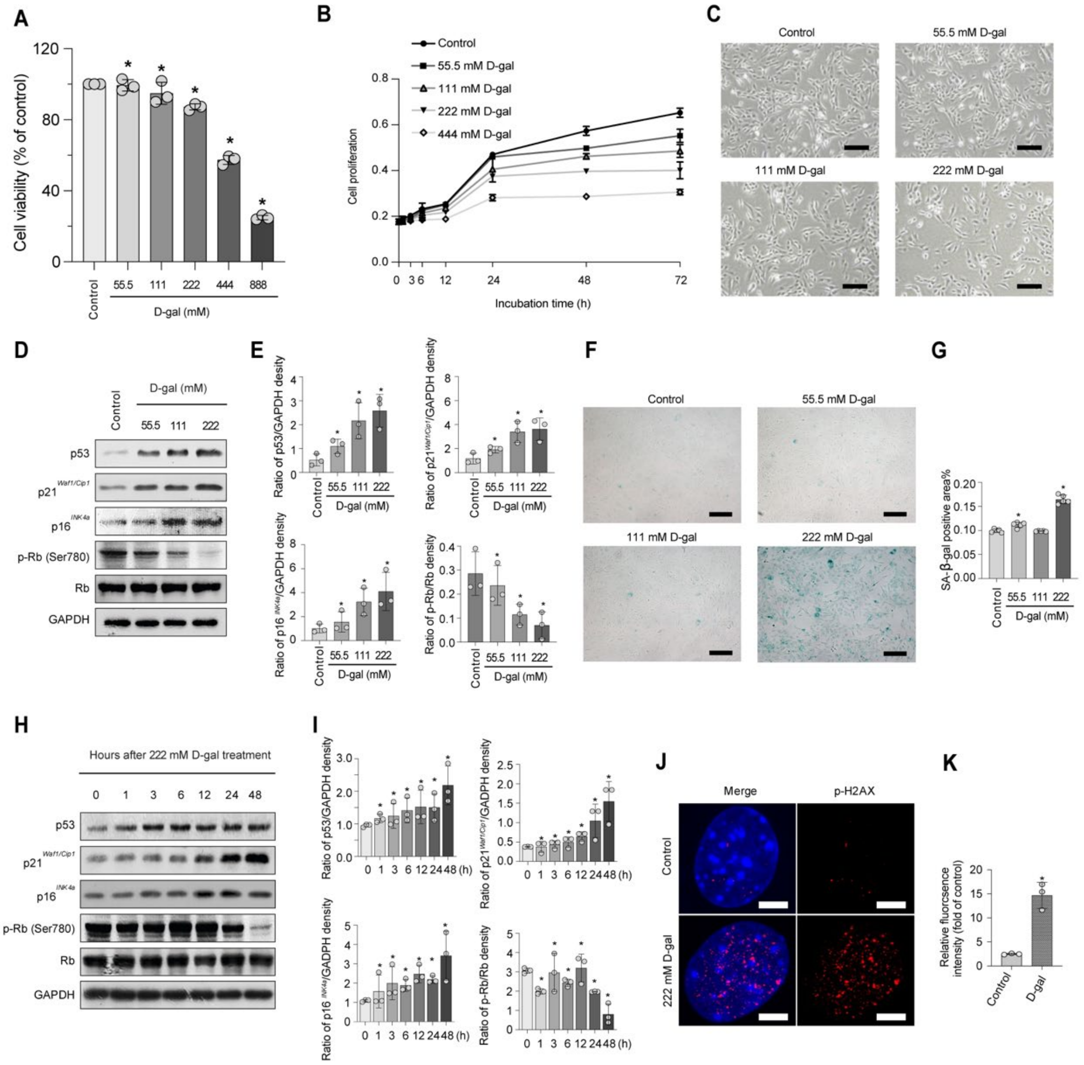

Figure S1. D-gal induces C2C12 myoblasts premature senescence.

(A, B) Cell viability and cell proliferation were examined by MTS assay. (C) Cell morphology was observed using light microscopy. Scale bar, 200 μm. (D) The protein expression was analyzed by western blotting. (E) Quantification of protein levels from (D). (F, G) Representative images of SA-β-gal staining of C2C12 myoblasts and SA-β-gal positive area%. Scale bar, 200 μm. (H) The protein expression was analyzed by western blotting. (I) Quantification of protein levels from (H). (J) Expression of p-H2A.X (red) was assessed by immunofluorescence staining, along with nuclear counterstaining using DAPI (blue). Scale bar, 5 μm. (K) Quantification of p-H2A.X levels from (J). Data are shown as the mean ± SEM from three independent experiments (n=3). $* P < 0.05$ versus control.
